# Molecular Linkage Mapping and Marker-Trait Associations with *NlRPT*, a Downy Mildew Resistance Gene in *Nicotiana langsdorffii*

**DOI:** 10.3389/fpls.2012.00185

**Published:** 2012-08-24

**Authors:** Shouan Zhang, Muqiang Gao, David Zaitlin

**Affiliations:** ^1^Kentucky Tobacco Research and Development Center, University of KentuckyLexington, KY, USA

**Keywords:** molecular markers, disease resistance, linkage mapping, AFLP, *Nicotiana benthamiana*

## Abstract

*Nicotiana langsdorffii* is one of two species of *Nicotiana* known to express an incompatible interaction with the oomycete *Peronospora tabacina*, the causal agent of tobacco blue mold disease. We previously showed that incompatibility is due to the hypersensitive response (HR), and plants expressing the HR are resistant to *P. tabacina* at all stages of growth. Resistance is due to a single dominant gene in *N. langsdorffii* accession S-4-4 that we have named *NlRPT*. In further characterizing this unique host-pathogen interaction, *NlRPT* has been placed on a preliminary genetic map of the *N. langsdorffii* genome. Allelic scores for five classes of DNA markers were determined for 90 progeny of a “modified backcross” involving two *N. langsdorffii* inbred lines and the related species *N. forgetiana*. All markers had an expected segregation ratio of 1:1, and were scored in a common format. The map was constructed with JoinMap 3.0, and loci showing excessive transmission distortion were removed. The linkage map consists of 266 molecular marker loci defined by 217 amplified fragment length polymorphisms (AFLPs), 26 simple-sequence repeats (SSRs), 10 conserved orthologous sequence markers, nine inter-simple sequence repeat markers, and four target region amplification polymorphism markers arranged in 12 linkage groups with a combined length of 1062 cM. *NlRPT* is located on linkage group three, flanked by four AFLP markers and one SSR. Regions of skewed segregation were detected on LGs 1, 5, and 9. Markers developed for *N. langsdorffii* are potentially useful genetic tools for other species in *Nicotiana* section *Alatae*, as well as in *N. benthamiana*. We also investigated whether AFLPs could be used to infer genetic relationships within *N. langsdorffii* and related species from section *Alatae*. A phenetic analysis of the AFLP data showed that there are two main lineages within *N. langsdorffii*, and that both contain populations expressing dominant resistance to *P. tabacina*.

## Introduction

The downy mildews are a group of biotrophic oomycetes that form parasitic, often species-specific interactions with certain groups of angiosperms. Commonly known as water molds, oomycetes are spore-forming filamentous protists that superficially resemble fungi, but actually share a common evolutionary history with the photosynthetic chromophyte algae, a group that includes diatoms, brown algae, and giant kelps in the kingdom Stramenopila (Kamoun et al., 1999; Petersen and Rosendahl, 2000; Dick, 2002; Tyler et al., 2006). Many oomycete species are familiar plant and animal pathogens, and several have historically caused devastating agricultural disease epidemics that resulted in widespread human suffering and significant economic losses. Among the well-known examples of this are the Great Famine in Ireland (1845–1852) caused by the potato late blight pathogen (*Phytophthora infestans*), and the grape downy mildew (*Plasmopara viticola*) epidemic of 1878–1882 that nearly destroyed the French wine industry (Johnson, 1935; Ristaino, 2002). The genus *Peronospora sensu latu* (*Peronosporales*) is the largest genus of downy mildews; all species are obligately biotrophic parasites that, as a group, infect a broad range of dicot species (families such as *Fabaceae, Chenopodiaceae, Rosaceae, Caryophyllaceae, Rubiaceae, Solanaceae, Papaveraceae, Euphorbiaceae* etc.), as well as a single family of monocots, the *Alliaceae*. Molecular systematic treatments based on the nuclear ribosomal internal transcribed spacer region (nrITS, including the 5.8S rRNA gene) have shown *Peronospora* to be a monophyletic group closely related to *Phytophthora* (Cooke et al., 2002; Voglmayr, 2003), a genus that also contains many destructive plant pathogens. In addition, species parasitic on cruciferous hosts (including *Hyaloperonospora*; Slusarenko and Schlaich, 2003) were found to group together in a highly supported clade that was sister to all other species of *Peronospora* (Voglmayr, 2003). More recent phylogenetic analyses based on additional rDNA and gene sequence data (coding and non-coding) from more downy mildew taxa confirmed the previous results and provided an evolutionary scheme for 14 genera of the *Peronosporales* and its closest relative, *Phytophthora* (Göker et al., 2007).

Genetic resistance to phytopathogenic oomycetes is well-known in dicotyledonous plants. There are many examples of single dominant genes conferring host resistance in major crop species, as well as in *Arabidopsis*, and some of these have been cloned and characterized (Mauch-Mani, 2002; Martin et al., 2003; Hein et al., 2009). Observed resistance phenotypes often involve the hypersensitive response (HR), a form of programmed cell death (PCD) in the host. In fact, the HR appears to be a common feature of host resistance to downy mildews and other oomycete pathogens in general (Kamoun et al., 1999; Vleeshouwers et al., 2000).

Wild relatives of crops such as lettuce, sunflower, tomato, and potato have proven to be valuable sources of genetic resistance to oomycete pathogens (Van Der Vossen et al., 2003; Lebeda et al., 2008; Wieckhorst et al., 2010). The most economically important of these genes provide resistance to late blight, caused by *P. infestans*, in commercial cultivars of potato (*Solanum tuberosum*). Resistance to *P. infestans* is known from at least eight wild potato species, with many of the single dominant genes for late blight resistance originating in two diploid species, *Solanum bulbocastanum* and *S. demissum* (Park et al., 2009). Similarly, *WRR4*, a dominant gene that conditions broad-spectrum resistance to important races of *Albugo* *candida*, was recently isolated and characterized from the model dicot *Arabidopsis thaliana* Col-0 (Borhan et al., 2008). *A. candida* is an obligately biotrophic oomycete pathogen that causes white blister rust on a number of species in the Brassicaceae. *WRR4* has been demonstrated to confer full white rust resistance to single susceptible cultivars of *B. napus* and *B. juncea* (Borhan et al., 2010), and it is possible that this and other white rust R-genes from *Arabidopsis* will be incorporated into commercial *Brassica* crops that are susceptible to infection by *A. candida*.

*Peronospora tabacina* D.B. Adam (syn. *P. hyoscyami* de Bary) is the causal agent of blue mold disease, a major foliar disease of cultivated tobacco in many areas of the world where tobacco is grown. *P. tabacina* can infect all types of tobacco at all stages of growth and can spread rapidly under favorable weather conditions due to the polycyclic nature of this intimate host-pathogen association (Main, 1991). Historically, blue mold has been known in the US since 1921, when it first appeared in tobacco seedbeds in Georgia and Florida, although it took 10 years for it to become a persistent problem (Wolf, 1947). Due to the intensity of asexual sporangia production on infected plants, the potential for epidemic development, and associated crop damage, can be very high when optimal environmental conditions for pathogen growth and sporulation prevail. Fungicides are generally applied in an attempt to limit economic losses caused by blue mold infection. The systemic phenylamide fungicide metalaxyl (Ridomil^®^; Syngenta Crop Protection, Greensboro, NC, USA), an inhibitor of rDNA synthesis, is effective for controlling *P. tabacina* in the field, and is also active against other foliar and soil-borne species of *Phytophthora* and *Pythium* in crops such as potato, grapes, and some ornamentals (Gisi, 2002). However, resistant strains of *P. tabacina* have developed in some countries as a result of excessive fungicide application (Wiglesworth et al., 1988; Main, 1991). Genetic resistance in the host (tobacco cultivars) would be the most economical and environmentally friendly means of controlling blue mold epidemics in the field.

Cultivated tobacco (*N. tabacum*) is an excellent host for *P. tabacina*, and no naturally resistant variety or cultivar has been identified, despite an intensive search (Clayton, 1945; Julio et al., 2006b). A number of Australian species of *Nicotiana* are highly resistant to *P. tabacina* infection; quantitative, polygenic resistance to blue mold has been transferred into tobacco from species such as *N. debneyi*, and several partially resistant tobacco cultivars were released in Australia and the United States beginning in the 1960s (Clayton, 1967, 1968; Rufty et al., 1990). Natural sources of monogenic resistance to several other tobacco diseases have also been identified in undomesticated species of *Nicotiana* and transferred to cultivated tobacco. Examples are the *N* gene for TMV resistance from *N. glutinosa* (Holmes, 1938; Dinesh-Kumar et al., 1995), the *Ph* gene for black shank resistance from *N. plumbaginifolia* (Johnson et al., 2002a), and monogenic dominant resistance to black root rot (*Chalara elegans*) from *N. debneyi* (Kenward et al., 1999). Host reactions to blue mold infection are dependent upon factors such as plant age, physiological status, and environmental conditions, and the results from field experiments can be highly variable and unpredictable. Thus, identification of molecular marker loci that are tightly linked to a blue mold resistance gene could be used to eliminate the susceptible individuals during early stages of a breeding program. Development of a molecular map would also make positional or map-based cloning of such resistance genes possible.

Molecular genetic markers have been developed for several species of *Nicotiana*, often with an emphasis on phylogenetic reconstruction and genetic distance estimation (Bogani et al., 1997; Yu and Lin, 1997; Ren and Timko, 2001; Julio et al., 2006b; Yang et al., 2007). In *N. tabacum*, PCR-based marker systems that do not rely on genomic sequence information have been widely used in both genetic studies and marker-assisted selection. Methods such as AFLP (Vos et al., 1995) and random amplified polymorphic DNA (RAPD), which employs short primers of arbitrary sequence (Williams et al., 1990), are well suited to marker discovery in species like tobacco that have large and complex genomes. RAPDs, often in concert with bulked segregant analysis (BSA; Michelmore et al., 1991), have been particularly useful for detecting marker loci linked to genes or genomic regions that confer resistance to tobacco diseases such as blue mold (Milla et al., 2005) and black root rot (Bai et al., 1995), both of which were introgressed into *N. tabacum* from *N. debneyi*, black shank resistance that originated in *N. plumbaginifolia* (Johnson et al., 2002a,b), and *potato virus Y* (PVY) resistance from *N. africana* (Lewis, 2005). AFLP markers closely linked to the *tomato spotted wilt virus* resistance locus from *N. alata* were identified by Moon and Nicholson (2007). A linkage map consisting of 171 restriction fragment length polymorphism (RFLP) and RAPD loci was constructed for the *N. longiflora/N. plumbaginifolia* genome by Lin et al. (2001). Partial molecular marker-based linkage maps for *N. tabacum* have also been reported by Nishi et al. (2003), who identified a quantitative trait loci (QTL) associated with bacterial wilt resistance, and by Julio et al. (2006a) in a study of QTL affecting a diverse variety of agronomic and smoking-related traits. A comprehensive linkage map of the *N. tabacum* genome, based on the segregation of 2317 simple-sequence repeat (SSR) markers in an F_2_ population, was recently published by Bindler et al. (2011). In addition, Wu et al. (2010) reported the construction of conserved ortholog set (COS)II- and SSR-based marker linkage maps for the *N. tomentosiformis/N. otophora* and *N. acuminata* (both *n* = 12) genomes.

We previously reported the discovery and initial characterization of a necrotic lesion resistance response to *P. tabacina* infection in *N. langsdorffii* Weinm. (Solanaceae, *Nicotiana* section *Alatae*), a diploid South American relative of cultivated tobacco (*N. tabacum* L.). In this host-pathogen interaction, *P. tabacina* resistance is mediated by the HR, is expressed in seedlings as well as adult plants, and is due to the presence of a single dominant gene called *NlRPT* in the host (Zhang and Zaitlin, 2008). By using BSA, several AFLP markers were identified that showed close linkage to the *NlRPT* locus. The objectives of this study were to determine the feasibility of using amplification-based molecular markers (AFLP, SSR, CAPS, inter-simple sequence repeat (ISSR), target region amplification polymorphism (TRAP), SCAR, and COS) to develop a molecular linkage map of the *N. langsdorffii* genome, and to identify marker loci associated with *NlRPT*. Both of these objectives were met, and we present a preliminary molecular marker-based map of the *N. langsdorffii* genome that includes *NlRPT*. A longer-term goal of this project will be to use closely linked molecular markers to introduce *NlRPT* into tobacco cultivars as a potential strategy to control tobacco blue mold disease in the field. *N. tabacum* (*n* = 24) and *N. langsdorffii* (*n* = 9) are not closely related – they are classified in two different sections of the genus, sections *Nicotiana* and *Alatae*, respectively (Knapp et al., 2004). The two species are sexually incompatible, and gene transfer may therefore require a transgenic strategy. We were also able to use cluster analysis of AFLP data to understand the genetic relationships within a group of *N. langsdorffii* collections that differ in their reactions to *P. tabacina*.

## Materials and Methods

### Plant material and population development

Seeds of *N. langsdorffii* accessions S-4-1 through S-4-10 were obtained from various sources and are given in Table 1 of Zhang and Zaitlin (2008). *Nicotiana alata* PI#555473 (KTRDC accession #S-1-4), *N. forgetiana* PI#555501 (KTRDC #S-11-2), and *N. longiflora* PI#555531 (KTRDC #S-33-1) were from the USDA Tobacco Collection. *N. longiflora* (KTRDC #S-33-5) was obtained from the IPK-Gatersleben as accession NIC44. MO-9, MO-10, and MO-11 are wild *N. langsdorffii* seed collections made in December 1999 in Santa Catarina state, Brazil, and were the gift of Dr. Tim Holtsford of the University of Missouri, Columbia (see Lee et al., 2008). MO-9 was found to be segregating for HR-mediated resistance to *P. tabacina*, and two sub-lines, designated MO-9R (resistant) and MO-9S (susceptible), were selected from plants grown from the original seed provided by the Holtsford lab. Seeds of an unknown species of *Nicotiana* collected near Caçapava do Sul in the state of Rio Grande do Sul, Brazil was the gift of Mr. Mauro Peixoto of Mogi das Cruzes, São Paulo, Brazil. Comparisons of mature plants grown from this seed for growth habit and floral and leaf characteristics with the description and illustration given in Goodspeed (1954) allowed us to identify it as *N. bonariensis* Lehmann, an uncommon member of *N*. section *Alatae*. The original source of *N. alata* (Jasmine tobacco), KTRDC accession #S-1-1, is unknown.

**Table 1:** **AFLP primers used for bulked segregant analysis (BSA), genetic linkage mapping, and cluster analysis in *N. langsdorffii***.

AFLP primer^1^	DNA sequence (5′ → 3′)
E01	AGACTGCGTACCAATTCA
M02	GATGAGTCCTGAGTAAC
E32	GACTGCGTACCAATTCAAC
E33	GACTGCGTACCAATTCAAG
E36	GACTGCGTACCAATTCACC
E39	GACTGCGTACCAATTCAGA
E40	GACTGCGTACCAATTCAGC
E41	GACTGCGTACCAATTCAGG
M47	GATGAGTCCTGAGTAACAA
M48	GATGAGTCCTGAGTAACAC
M49	GATGAGTCCTGAGTAACAG
M50	GATGAGTCCTGAGTAACAT
M51	GATGAGTCCTGAGTAACCA
M52	GATGAGTCCTGAGTAACCC
M53	GATGAGTCCTGAGTAACCG
M54	GATGAGTCCTGAGTAACCT
M55	GATGAGTCCTGAGTAACGA
M56	GATGAGTCCTGAGTAACGC
M57	GATGAGTCCTGAGTAACGG
M58	GATGAGTCCTGAGTAACGT
M59	GATGAGTCCTGAGTAACTA
M60	GATGAGTCCTGAGTAACTC
M61	GATGAGTCCTGAGTAAGTG
M62	GATGAGTCCTGAGTAAGTT

*^1^All “E” primers were labeled at the 5′ end with WellRed D2 dye for fluorescent detection*.

*N. langsdorffii* accession S-4-1 is highly susceptible to infection and colonization by *P. tabacina*, while accession S-4-4 is characterized as being highly resistant, developing HR necrotic lesions 2 days after inoculation with the pathogen (Zhang and Zaitlin, 2008). Ninety-two progeny from the “modified backcross” population BC1-1 [(S-4-1 × S-4-4) F_1_ × *N*. *forgetiana* S-11-2] were used to construct the linkage map. For BSA, DNA from progeny of an F_2_ population (derived from the S-4-1 × S-4-4 F_1_) was used to construct two susceptible (S) bulks. A description of the segregating populations, as well as methods for pathogen inoculation, disease ratings, and plant reactions are given in Zhang and Zaitlin (2008).

### DNA extraction

Genomic DNA was prepared from expanding leaves of young plants using the DNeasy Plant Mini Kit (QIAGEN Inc., Valencia, CA, USA) according to the manufacturer’s instructions, and was quantified with a NanoDrop ND-1000 spectrophotometer (NanoDrop Products, Wilmington, DE, USA).

### AFLP reactions

AFLP manipulations were performed as described by Vos et al. (1995) using an AFLP Core Reagent Kit from Invitrogen (Carlsbad, CA, USA). Fluorescent Eco+3 primers labeled at the 5′ end with the WellRed D2 dye (Beckman Coutler) were synthesized by Proligo LLC (Boulder, CO, USA). Unlabeled primers were purchased from Integrated DNA Technologies (Coralville, IA, USA). Genomic DNA samples (∼0.2 μg) were digested with *Eco*RI and *Mse*I at 37°C in a final volume of 25 μl. Following ligation of the *Eco*RI and *Mse*I- specific adaptor sequences at 20°C overnight (15 h), the reactions were diluted 10-fold with TE (10 mM Tris-HCl, 0.1 mM EDTA, pH 8.0). Pre-selective amplifications (PSAs) were performed in 25 μl of 1× FailSafe “A” premix (Epicenter Biotechnologies, Madison, WI, USA) with E01 and M02 primers (with a single 3′ selective base) at 0.5 μM, Taq DNA polymerase (New England Biolabs, Beverly, MA, USA) at 1 U per reaction, and 3 μl of diluted ligation DNA for 22 cycles (94°C for 30 s, 56°C for 60 s, and 72°C for 60 s). The PSA reactions were examined by electrophoresis on a 1.5% agarose gel for the presence of a predictable band pattern before proceeding. For selective amplification (SA), PSA reactions were diluted 20-fold in deionized water and amplified with E+3 and M+3 primers (three selective bases at the 3′ end) using a “touchdown” cycling program (Vos et al., 1995) consisting of an initial denaturation step of 94°C for 2 min, followed by 10 cycles of 94°C for 20 s, 66°C for 30 s, and 72°C for 2 min with the annealing temperature decreased by 1°C/cycle, and then 20 cycles of 94°C for 20 s, 56°C for 30 s, and 72°C for 2 min with a final step of 30 min at 60°C. In a total volume of 20 μl, each SA reaction contained 10 μl FailSafe “A” premix (Epicenter), 1.5 pmol dye-labeled E+3 primer, 6.25 pmol unlabeled M+3 primer, 1 U Taq DNA polymerase, and 4 μl of diluted PSA DNA. All DNA amplifications were performed in an iCycler Thermal Cycler (Bio-Rad Laboratories, Hercules, CA, USA). Following amplification, SAs were diluted 30-fold into Sample Loading Solution (SLS; Beckman Coutler, Fullerton, CA, USA) containing a 100-fold dilution of DNA Size Standard-600 (Beckman Coulter). Amplified fragments were separated by capillary electrophoresis on an automatic DNA sequencing instrument (Beckman Coulter CEQ8000 Genetic Analysis System) using the Frag-4 method. For each primer combination, sample order was randomized on the CEQ8000 to avoid any positional effects due to potential variations in the individual capillaries. The AFLP primers used are shown in Table 1. Primer nomenclature is from KeyGene N.V.^1^.

### AFLP data analysis

DNA fragment peak sizes in the D2 channel were calculated (in nucleotide bases) against the standards (60–640 bases) in the D1 channel using the software supplied with the CEQ8000 (Beckman Coulter). In our hands, the quartic equation gave the best approximation of a linear relationship between peak size and migration time with the 600 standard. For genotypic cluster analysis, fluorescent peaks were machine scored (1 = present, 0 = absent) using the “New AFLP Analysis” module (parameter settings: 15% slope threshold, 5% relative peak height threshold, 95% confidence level, bin width = 1 bp) and the data was exported to Microsoft Excel. Every AFLP chromatogram was examined for the presence of unscored peaks at a minimum height threshold of 500 fluorescence units. Data entries from poorly resolved and miscalled peaks were manually removed from the spreadsheets. Bins containing peaks outside of the standard size range (<60 and >640 bases) were eliminated. All manual scoring was performed by the same individual to ensure consistency.

### Bulked segregant analysis and AFLP markers

Two DNA bulks (S#1 and S#2) were prepared by pooling approximately equal amounts of total DNA from 12 and 15 blue mold susceptible F_2_ progeny plants, respectively, that were selected based on their responses to blue mold infection (Table 3 in Zhang and Zaitlin, 2008). Pooled DNA samples to be used for amplification were adjusted to a concentration of 100 ng/μl, and 500 ng of each was double digested with *Eco*RI and *Mse*I and prepared for AFLP analysis as described above. The S#1 and S#2 bulks, the two *N. langsdorffii* inbred lines (S-4-1 and S-4-4), the F_1_ parent (S-4-1 × S-4-4), and *N. forgetiana* S-11-2 were screened with a total of 46 AFLP primer pairs (Tables 1 and 2) in an effort to identify amplified peaks showing an association with the *NlRPT* gene. Based on evaluations of these six DNA samples, 92 individuals from the modified BC population, the three parental accessions (S-4-1, S-4-4, and S-11-2), and the F_1_ were subjected to fluorescent AFLP analysis as described above with 14 pre-screened primer pair combinations (E32M47, E32M60, E33M58, E32M62, E33M48, E39M51, E39M52, E39M55, E39M59, E39M60, E40M50, E40M60, E41M48, and E41M50).

**Table 2:** **AFLP primer pairs used in BSA screening for linkage to the R-gene *NlRPT***.

AFLP primer pair	AFLPs linked to *NlRPT*
E32M47	None detected
E32M48	None detected
E32M56	None detected
E32M57	None detected
E32M58	None detected
E32M60	None detected
E32M62	E32M62_177
E33M48	E33M48_292
E33M52	None detected
E33M54	None detected
E33M55	None detected
E33M56	None detected
E33M58	None detected
E33M59	None detected
E33M60	None detected
E33M62	None detected
E36M47	None detected
E36M49	E36M49_261
E36M50	None detected
E36M51	None detected
E36M62	None detected
E39M48	None detected
E39M50	None detected
E39M51	E39M51_224
E39M52	None detected
E39M54	None detected
E39M55	E39M55_129
E39M56	None detected
E39M59	None detected
E39M60	None detected
E40M47	None detected
E40M49	None detected
E40M50	None detected
E40M51	None detected
E40M53	None detected
E40M54	None detected
E40M60	None detected
E40M61	None detected
E41M47	None detected
E41M48	None detected
E41M50	E41M50_198
E41M52	None detected
E41M55	None detected
E41M56	None detected
E41M58	None detected
E41M60	None detected

### Simple-sequence repeat markers

Genomic DNA of *N. langsdorffii* S-4-5, purified on a CsTFA gradient (Zhang et al., 2003), was provided to Genetic Identification Systems (GIS; Chatsworth, CA, USA) for the construction of plasmid-based genomic libraries enriched in DNA fragments containing di-, tri-, or tetranucleotide repeat sequences. Four such libraries, selected independently for CA/TG, GA/TC, AAC/GTT, and TAGA/TCTA repeats (libraries A, B, C, and D, respectively), were delivered in under 5 weeks from receipt of the DNA. DNA sequencing of several dozen plasmid clones was performed at GIS and KTRDC, but the majority (282) were sequenced at the University of Kentucky Advanced Genetics Technology Center. We were able to design oligonucleotide primers flanking the repeated sequence blocks for 98 SSR containing clones using Primer3 v. 0.4.0^2^. All primers were purchased from IDT^3^ (Coralville, IA, USA).

To identify polymorphic SSR markers, each primer pair was screened for amplification over a set of seven *Nicotiana* genotypes that included two accessions each of *N. langsdorffii*, *N. alata*, and *N. longiflora*, and *N. forgetiana* S-11-2. Amplified fragments were visualized and sized electrophoretically on gels made of 2% (w/v) MetaPhor^®^ agarose (Cambrex Bio Science Rockland, Inc., Rockland, ME, USA) in 0.5 × Tris-borate EDTA buffer (Amresco, Solon, OH, USA) containing 0.5 μg/ml ethidium bromide. Primer sequences and amplification conditions for the 29 SSR markers used here are given in Table 3.

**Table 3:** Primers for SSR marker amplification in *Nicotiana* spp., the *NlRPT* mapping population, and N. benthamiana*.

Locus name		*T*_anneal_ (°C)	DNA sequence (5′ → 3′)
GIS_A4*	F	55	TGCGCCAGTCAAATTCATACG
	R		GGAGTCCGCAAGAGAGGAATA
GIS_A6	F	54	TGGTGAAACAGTTGCCACAT
	R		AGTCGCCTCAAGACTGAAACA
GIS_A9	F	53	AACCCATGACAACGCATACA
	R		TGGTCTATTTGCCACGTGAA
GIS_B3*	F	55	AGTCTATGCGAGGGGACCTT
	R		TTGGTCCTGTGACTCCAACA
GIS_C12*	F	53	AAGAAATGGACAAACCAACTGC
	R		CCTTGTTTGGGACTGAAACGTA
GIS_D1*	F	54	GATGCAGACCAGTTGCTGAA
	R		TCAACCAACGCTGCAAGTAG
GIS_D3	F	52	GCAGTTGTATATTTGAAAGCCAAA
	R		TGCTGAAGGGTTAGATGTTCC
GIS_D7	F	52	TCTTTTGGGCTTTCCACTTTTA
	R		CTCGTGCCACAATATCATCAAT
KTRDC_B3	F	54	GTGCAAACTGAACTGGCTGAC
	R		TCAACATGGCTCGATAGATGG
KTRDC_B4	F	53	TATGGCAATGTGCTTTGTATGC
	R		TGCCATCATGAGATGTTTTTCA
KTRDC_B5*	F	53	CAAGCCATCCCATCTCCATA
	R		GCCAGTGAGGACAAACGAGT
A104	F	54	GGACAAAGTTAAGCCTACCCC
	R		GGACTCCAATCGCTGTATCAA
A112*	F	50	CAATTACGATTTCTTTCCATCG
	R		AAGTCACCTTGTTTGGTGTACG
A113*	F	53	GGACCATCTCAACAAAGATATGC
	R		TGTTACAGTTTTGTGGTTTAGGG
A118*	F	53	GCATTTTCCCAAGAGACAGACTA
	R		YTGGAAAGAAAAGAAGATGAGCA
A152	F	56	TCCACAGCACAGACCCTTTACT
	R		CTCACACACAAACACACACACG
A154	F	54	TATCACCAACGCACTCATCTC
	R		AGTCAGGCTTGTCAGCTCATT
A159*	F	52	CCCATAAAATTGGGGCCTTA
	R		TGGAGTTGATGGTTGTTCCTG
A160	F	55	ATGACGAGTGCCTGAGTCCTA
	R		GCGTGTGACCTTATCAGCTTC
A161*	F	53	CCGCTAGTTGAATCCTCAATTCT
	R		TGATATGATTACCCCTGGTGTTG
A172	F	53	CACGTATTTGCCTGCCTAACA
	R		CAAGGTCCCATATTGGGCTAA
A204*	F	54	CTTGGTCAACGGGTTCATCT
	R		ATGTGGAACTGCGGTTATGC
A207	F	53	TATTTCACCCACAGCCTTCTT
	R		GTGTTCACCTGTGCCAATTAAA
A218*	F	54	TCGGCCTCACAGCAATAATC
	R		CTCATCTCCCAGGCCCTAAA
A224*	F	55	CAGAGGATCATGCGACCACT
	R		CCGCCTCTTGCACAAAGTAG
B151	F	54	CCTTCCCTACTCAAGTTTCAATGT
	R		AAACAGAGAACGCCAAATGGA
B155*	F	55	GTCGAGAGGGCTAAGTGAACAA
	R		ACAAGTCATCGACACGCGACAA
B158	F	54	AAGCCTTTTCTTTTTGAGGGACT
	R		CTTCAATCTTCAAACCACCACTG
B162*	F	55	TGAGAAGGGGAAAAGGAGGTT
	R		TACTCTCAGCGAGGTGACGAA
B202*	F	52	CAAGGTCTGCTATTGTGAGTG
	R		CACCAGTGTTCCACGTAAAGAA
B211*	F	54	TGCTGTTGCAAGGTCTGCTAT
	R		AAATCTGGAGTGCCCAATGTC
B215*	F	55	CCCAGTCTCCTTAGCCATCTG
	R		AGGGCAGCATATCCGCTACT
C156*	F	53	CACAATATCCTGCCCATACCC
	R		GAAGAAATTCCCGGCTTCAC
C157	F	53	TCACTATTCTTGAGCCGAGGA
	R		GTCATTCCTGTCGATTGATGC
C161	F	53	TCACAAGATGGAACAGTACATGC
	R		TCCAAAGACTTTTGTAGTGGTGA
C167	F	54	CCTTGAGCCGAGGGTCTATT
	R		GGGCAGGCAATTGAGATACA
C202*	F	54	TGGGATCACACTGGGTATGT
	R		TCCATTCGGGTTACCTTTTCC
D152	F	53	GCGAACGACATTTCGCTAAG
	R		ATAGGGAACACGCGCAACAT
D159	F	54	AATACCGAACCGAAATACCGAAT
	R		AAGCGAACGACATTTGACTAAGG
D161	F	54	GATGCAGACCAGTTGCTGAA
	R		TCAACCAACGCTGCAAGTAG
D171	F	54	TCGCATGAAAGAGACCTCGTA
	R		CTCCGAAGGAACACATCCATT

### Conserved ortholog set markers

Primer sequences for 51 COSII markers (Fulton et al., 2002; Mueller et al., 2005; Wu et al., 2006) previously mapped in tomato were obtained from the SOL Genomics Network (SGN) website^4^, and the oligonucleotides were synthesized by IDT. In all cases, primer pairs were screened for amplification against a panel of six genomic DNA samples; *N. obtusifolia* PI555573, *N. benthamiana*, Roma tomato, *N. langsdorffii* S-4-1 and S-4-4, and *N. forgetiana* S-11-2. We used a single PCR regimen and assayed each primer pair in both FailSafe pre-mixes “A” and “D” (a total of 12 amplifications per screening panel). Twenty-one primer pairs directed amplification of a discreet fragment from at least one of the species assayed. Those that gave single fragments from S-4-1, S-4-4, and S-11-2 were subjected to further evaluation as CAPS markers (Konieczny and Ausubel, 1993). The fragments from the three parental accessions were individually digested with up to 12 restriction endonucleases (*Alu*I, *Ava*I, *Bfa*I, *Dpn*II, *Hae*III, *Hin*fI, *Hin*P1I, *Mse*I, *Msp*I, *Rsa*I, *ScrF*1I, and *Taq*I) in an attempt to identify an enzyme that allowed us to unambiguously distinguish the S-4-1 and S-4-4 allelic fragments from one another, without interference from the S-11-2 PCR product. Primer sequences for the COSII markers and the restriction enzymes used in this study are given in Table 4.

**Table 4:** **Primers used for PCR amplification of COSII, SCAR, ISSR, and TRAP markers in *N. langsdorffii***.

Marker locus	Type		*T*_anneal_ (°C)	Restriction enzyme	DNA sequence (5′ → 3′)
At1g02140	COSII	F	55	*Hin*FI	TCCGTTATGCTAACAATTCCAAC
		R			TGTGTTCATTTCCCATCACAATCTC
At1g30580	COSII	F	54	*Scr*FI	TTCTGCCGAAGATTCATGCATGG
		R			TCTCTCCACAGCAGCACTGAAAGG
At1g60640	COSII	F	53	*Dpn*II	TCTGACAGGGGGAAATGAATCTTC
		R			ACTTCATTGAAAGAGCTATCTTCACTCTC
At2g16920	COSII	F	55	*Taq*I	TGCAAGACAATGATGTATCTGATGAG
		R			TCCTAAAGTGCTCTCTGATAAGCTCT
At3g10220	COSII	F	54	None	TGGCTTCTCAGTTACAGATTCAAGG
		R			AACCTCCGGAGGTCACTGACG
At4g21520	COSII	F	52	None	TGACGGGGAGTCTGTATATGACTATTG
		R			TGATTGCCCTTCTTTATTTCCTTG
At5g37360	COSII	F	52	*Dpn*II	TGCAGCATTTGATCTTTCAAATGG
		R			TGATTTTTGAGAGCCTGTCAATGAG
At5g62390	COSII	F	54	*Rsa*I	TGCTACTAACTGTTGATGCCATTGAG
		R			TTGGGGGTCGATAACATCAAGC
At5g64350	COSII	F	53	*Ava*I	AGATCGGCCAAGGCAAAGTTATC
		R			TGCATGCCCAGTACTCCTTCATCC
UBC825	ISSR		49	None	ACACACACACACACACT
UBC826	ISSR		51	None	ACACACACACACACACC
U826_900	SCAR	F	53	None	ACACACACACACACACCAAAAGG
		R			ACACACACACACACACCACTCTC
U826_1400	SCAR	F	54	*Dpn*II	ACACACACACACACCCAGATTG
		R			ACACACACACACACACCAGAAC
U826_1900	SCAR	F	55	None	ACACACACACACACACCGTGT
		R			ACACACACACACACACCAAAAAA
NBS9^1^	TRAP		50		TGTGGAGGRTTACCTCTAGC
Em3					GACTGCGTACGAATTGAC
ME2					TGAGTCCAAACCGGAGC

*^1^Primer labeled with WellRed dye D3 at the 5′ end to enable fluorescent detection*.

### ISSR and SCAR markers

The two *N. langsdorffii* genotypes (S-4-1 and S-4-4) and *N. forgetiana* S-11-2 were screened with 7 ISSR primers (UBC817, 819, 825, 826, 827, 849, and 850) from the University of British Columbia set #9 (see Sankar and Moore, 2001) for amplified bands that were unique to either of the *N. langsdorffii* parents and also absent in *N. forgetiana*. We observed that some ISSR fragments amplified inconsistently in the mapping population. Consequently, ten DNA fragments (five generated with UBC826 and five generated with UBC827) were purified by agarose gel electrophoresis and cloned into pGEM-T (Promega). The recombinant plasmids were digested with *Bst*ZI (Promega) to size the inserted DNA fragments, and selected plasmids were subjected to DNA sequencing. The original ISSR primers (17 bases in length) were converted to SCAR (sequence characterized amplified region) marker primers by extending the 3′ ends with 4–7 selective bases (Albani et al., 2004). The SCAR primer sequences are given in Table 4.

### SRAP and TRAP markers

Target region amplification polymorphism markers (Hu and Vick, 2003) were amplified using the gene-specific primer NBS9, labeled at the 5′ end to enable fluorescent detection, and unlabeled random primers from Li and Quiros (2001). NBS9 was designed to be complementary to the conserved GLPL motif of the nucleotide binding site domain found in many plant R-genes (Wang et al., 2008). Amplification reactions (20 μl) contained 1× FailSafe “A” premix (Epicenter), 10 pmol of each primer, and ∼50 ng genomic DNA. Amplification parameters were the same as given for SRAP markers (Li and Quiros, 2001).

### Data scoring and linkage analysis

The segregating population (which we have called a “modified” BC_1_) was designed so that all polymorphic markers, regardless of type (AFLP, SSR, COS, CAPS, etc.) could be scored using a single common format; the S-4-1 allele was designated “a” and the S-4-4 allele was designated “b” for each segregating locus. Allelic data scores for each individual were entered manually into a spreadsheet, which was then converted to a simple text file for analysis by JoinMap 3.0 (licensed copy provided by H. Zhu). The data was analyzed using the following parameters: HAP1 population type; maximum recombination frequency of 0.45; initial LOD threshold of 4.0 for the identification of linkage groups (LGs); LOD threshold of 6.0 for the final linkage group determination. The Haldane mapping function was employed. Graphical maps were generated with MapChart version 2.2 (Voorrips, 2002) from text files exported from JoinMap.

### Cluster analysis of *N. langsdorffii* accessions

Fluorescent AFLP peak data was collected for 14 accessions of *N. langsdorffii*, and a single accession each of *N. alata, N. forgetiana, N. longiflora*, and *N. bonariensis*. We used six AFLP primer pairs (E+3/M+3), which gave a total of 1300 unique fragment bins for the set of 18 entries. A dissimilarity matrix was calculated using NTSYSpc 2.2 (SIMINT with the DIST option selected; Rohlf, 2007) so that the AFLP data could be clustered using the Neighbor-Joining (N-J) algorithm (Saitou and Nei, 1987; Kim et al., 2004). Bootstrap analysis was also performed with NTSYSpc 2.2 by using the program modules RESAMPLE to generate 1000 resampled files of the AFLP data, SIMINT to calculate the distance matrices, NJOIN to calculate the N-J trees, and CONSENS (with MAJRUL enabled) to compare the trees and calculate the cluster frequencies. The cophenetic correlation coefficient (*r* = 0.96086, normalized Mantel statistic *z*) was calculated using the COPH and MXCOMP modules in NTSYSpc. Principal coordinates analysis (PCoA) was also performed using the statistical software package MVSP 3.2 (Kovach Computing Services, Anglesey, Wales, UK). AFLP data was imported in spreadsheet format, and the following program settings were used; data matrix transformed, Euclidean distances computed, eigenanalysis tolerance set to 10^−7^. The resulting graphs were exported as enhanced metafiles, and text labels were added with the program Metafile Companion (Companion Software, Sunderland, MA, USA).

## Results

### Segregating population structure and marker development

In our *N. langsdorffii* population BC1-1 (the modified backcross), necrotic lesion resistance to *P. tabacina* infection segregated in the expected 1:1 ratio (Zhang and Zaitlin, 2008). Ninety-two individuals from this progeny set were chosen at random to constitute the genetic mapping population. All molecular markers used in this study were PCR-based, and, with the exception of the COS markers, were developed specifically for *N. langsdorffii*. Of the 98 SSR primer pairs designed from the genomic clones, 89 gave amplification products in at least one of the species of *Nicotiana* assayed, and 30 gave amplification products that differed in length between *N. langsdorffii* S-4-1 and S-4-4. Eleven of the SSR primer pairs gave amplification products only in *N. langsdorffii*, and five of these only gave a product from S-4-1 genomic DNA (S-4-1 is very similar to S-4-5, which was the DNA source used in the SSR enrichment). Fifty-nine of the SSR primer pairs gave amplification products from *N. alata* DNA, and 13 of these were polymorphic between the two accessions screened (S-1-1 and S-1-4). Similarly, 58 primer pairs directed amplification from genomic DNA of *N. longiflora*, and 20 gave length polymorphisms between accessions S-33-1 and S-33-5.

### Identification of AFLP markers linked to *NlRPT*

*NlRPT* is a dominant gene, and we did not observe an intermediate resistance phenotype in the F_1_ (S-4-1 × S-4-4). Because of this, we could not distinguish F_2_ progeny plants that were homozygous at this locus (*NlRPT/NlRPT*) from those that were heterozygous (*NlRPT/nlrpt*) by their disease resistance phenotypes. Following inoculation with *P. tabacina* sporangia, necrotic lesions developed at the infection sites at 48 hpi on all plants expressing blue mold resistance. Therefore, we could not use a resistant bulk for the AFLP marker screening. We screened two independent susceptible F_2_ bulks (S#1 and S#2) with 46 AFLP primer pairs to identify peaks that showed linkage to *NlRPT*. We examined peaks in approximately 4500 size bins, and found five peaks produced by five different primer pairs that met the following criteria: they were present in the resistant parent S-4-4 and the F_1_ but were absent in susceptible parent S-4-1, the S#1 and S#2 bulks, and *N. forgetiana* S-11-2. The identified peaks were E32M62_177, E33M48_292, E39M51_224, E39M55_129, and E36M49_261. AFLP nomenclature followed that of Jeuken et al. (2001), where marker names were determined by the primer pair abbreviation (Table 1) followed by the calculated size of the fluorescent peak in nucleotides.

### Linkage map construction

In our mapping population, we obtained allelic scores for 320 molecular markers that were polymorphic between S-4-1 and S-4-4. The population was structured so that all markers could be scored in the same manner with the expectation of a 1:1 allelic segregation ratio. AFLP, ISSR, and TRAP markers give amplification products from only one of the N. *langsdorffii* parents, and are analogous to genetic loci with dominant effects. Thus, we scored both the apparent (amplified) alleles and the null alleles for these markers. The progeny segregating for *P. tabacina* resistance came from the cross [(A_1_ × A_2_) × B], where A is *N. langsdorffii* (1 = S-4-1 and 2 = S-4-4) and B is *N. forgetiana* S-11-2. We scored marker alleles that were present in *N. langsdorffii* but absent in *N. forgetiana*. Because chromosomal assortment and inter-locus recombination occurred in the *N. langsdorffii* F_1_ parent (A_1_ × A_2_), the mapping progeny are genetically equivalent to a first generation backcross even though they are technically F_1_ hybrids between *N. langsdorffii* and *N. forgetiana*. Data analysis and map construction were performed with JoinMap 3.0 (Van Ooijen and Voorrips, 2001). Of the original 92 progeny, two (#s 18 and 39) were eliminated because fewer than 80% of the markers were scored in these individuals. The final data set consisted of 28,101 allelic scores from 90 individuals (2.43% missing data). The data for 29 AFLP loci that showed significant deviation from the expected 1:1 segregation ratio were eliminated from the analysis.

**Figure 1:**
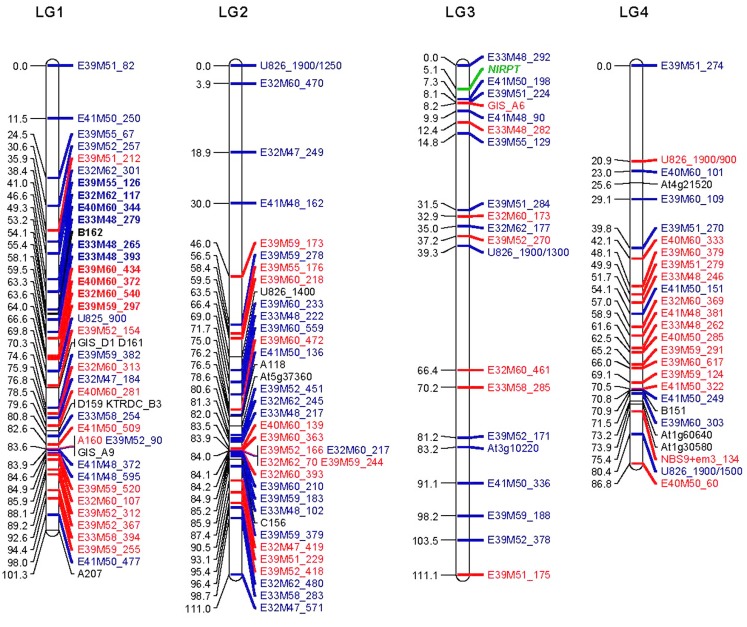

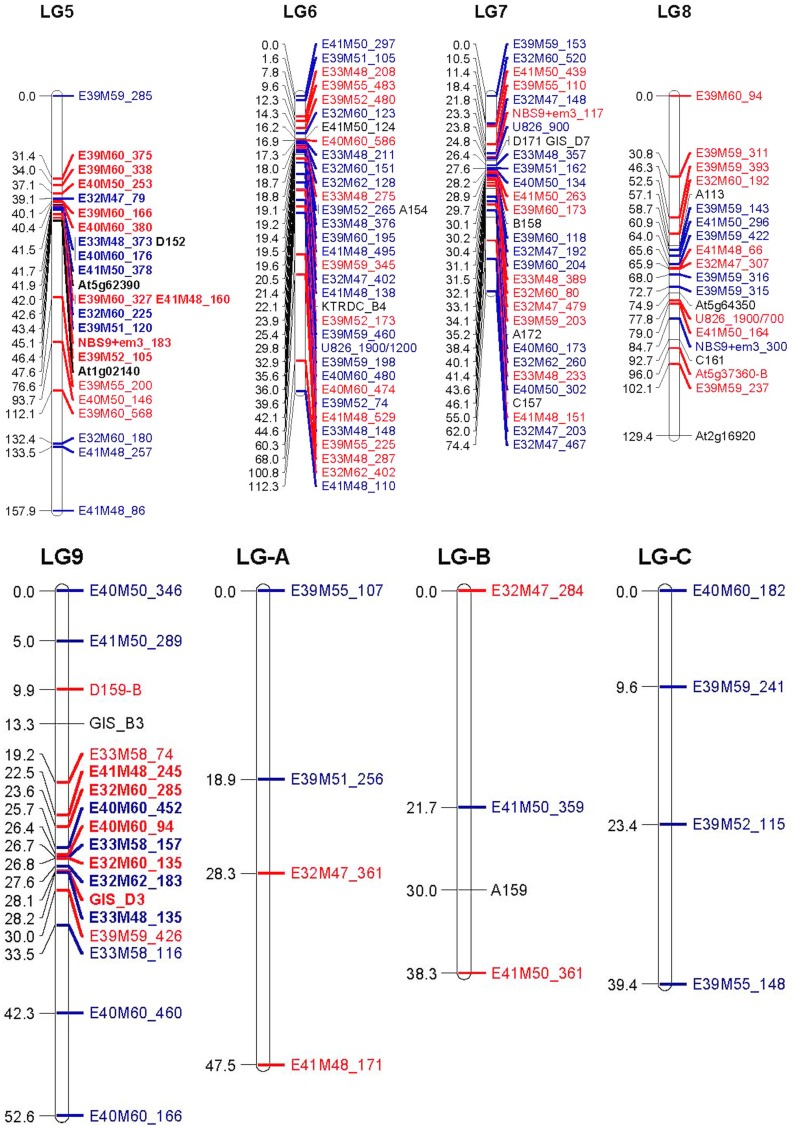
**Molecular marker linkage map of the *Nicotiana langsdorffii* genome**. The 12 linkage groups (LGs 1–9 plus LG-A, -B, and -C) together comprise a recombinational length of 1062 cM. The vast majority of the markers were present in only one of the two parental inbred lines used in the segregation studies. Loci indicated in red originated in S-4-1 (susceptible to *P. tabacina*), while those in blue came from S-4-4 (resistant to *P. tabacina*). Markers with alleles present in both S-4-1 and S-4-4 are shown in black. The R-gene locus, *NlRPT*, is shown in green. Locus names in the three segregation distortion regions (SDRs) on LGs 1, 5, and 9 are shown in boldface. Locus nomenclature: AFLPs – primer combination followed by fragment size in bases (e.g., ExxMxx_123); SSRs – locus names begin with A, B, C, D, GIS, or KTRDC; COSII – AtXg followed by a five digit number; ISSR loci – U825 or U826 followed by the fragment in bases; ISSR/SCAR – cloned fragment size indicated as a four digit number, followed by the size of the scored fragment if different (e.g., U826_1900/700); TRAP loci – gene-specific primer+random primer followed by the fragment size in bases (e.g., NBS9+em3_117).

The resulting map of the *N. langsdorffii* genome is shown in Figure 1. JoinMap grouped 266 molecular marker loci and the resistance gene *NlRPT* into 12 LGs. The LGs ranged from 38 to 158 cM in length, with a cumulative length of 1062 cM. A LOD threshold of 6.0 was used to calculate all LGs with the exception of LG5; reducing the LOD threshold to 5.0 resulted in the inclusion of a linked group of six additional loci, and maximized the length of this LG. Locus nomenclature for Figure 1 is as follows: amplified fragment length polymorphisms (AFLPs) – primer combination (ExxMxx) followed by the fragment size in nucleotide bases; SSRs – all start with A, B, C, D, GIS, or KTRDC; COSII markers – AtXg followed by a five digit number; ISSR loci – U825 or U826 followed by a three digit number indicating the size of the mapped fragment in bases; ISSR/SCAR markers have a similar format, with the cloned fragment size indicated as a four digit number, followed by the size of the scored fragment if different (e.g., U826_1900/700); TRAP loci – gene-specific primer+random primer followed by the fragment size in bases (e.g., NBS9+em3_117). In all cases, loci shown in black are co-dominant; dominant markers in red originated in the S-4-1 parent and those in blue originated in S-4-4; the position of *NlRPT* is indicated in green.

The haploid genome of *N. langsdorffii* consists of nine chromosomes, and a complete molecular marker map would be expected to have a corresponding number of LGs. In the map presented here, the nine largest LGs (LG1 through LG9) consist of between 18 (LG9) and 42 (LG1) loci (53 and 101 cM, respectively) with a cumulative length of 937 cM. There were also three LGs of only four marker loci each (LG-A, LG-B, and LG-C; 11 AFLPs, and one SSR) that had a combined length of 125 cM (11.8% of the total map distance). Twenty-four markers (7.5%) did not show strong linkage to any LG, and they were excluded from the analysis. *NlRPT* is located in a distal region of LG3 (111 cM), flanked by 4 AFLP loci and one SSR (GIS_A6) that together define a ∼10 cM region of this linkage group.

### Cluster analysis in *N. langsdorffii* and *Nicotiana* section *Alatae*

**Figure 2:**
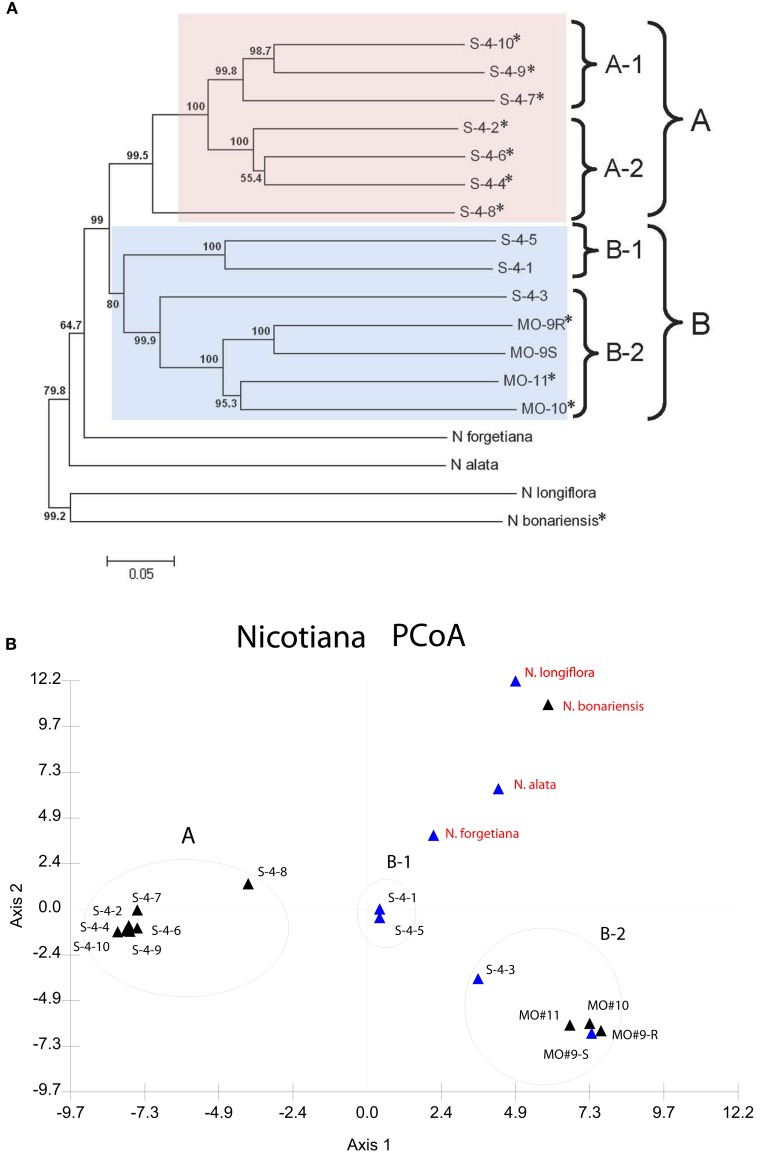
**(A)** Neighbor-joining (N-J) tree showing the relationships between 14 accessions of *N. langsdorffii* and four other species in *Nicotiana* section *Alatae*. Bootstrap values (1000 iterations) are shown adjacent to the nodes. Clusters A (pink) and B (blue) define the two lineages of *N. langsdorffii*; subclusters A-1, A-2, B-1, and B-2 are indicated by brackets. Accessions with an asterisk (*) displayed an incompatible interaction (HR) when infected by *P. tabacina* and are therefore resistant to this pathogen. **(B)** Principal coordinates analysis (PCoA) of the AFLP data for *Nicotiana* section *Alatae*. The first two orthogonal axes explain 34.183% of the total variation. Clusters A, B-1, and B-2 correspond to the clusters present in the N-J tree. All accessions of *N. langsdorffii* are labeled in black, while the other species in sect. *Alatae* are shown in red. Accessions that are either susceptible or resistant to *P. tabacina* infection are indicated by blue and black triangles, respectively.

A distance matrix derived from the binary AFLP data generated with six primer pairs was clustered using the N-J algorithm. The resulting tree (Figure 2A) shows the phylogenetic relationships within the group of 14 *N. langsdorffii* accessions, and between *N. langsdorffii* and four other related species in section *Alatae*. Bootstrap support values based on 1000 iterations are >50% for all nodes; only 1 (the S-4-4/S-4-6 pairing) is <60%, and 12 out of 16 are >95%. In this analysis, *N. langsdorffii* is monophyletic and is sister to *N. forgetiana*, although the 14 accessions of *N. langsdorffii* are distributed between two major clusters (A and B), both of which show some substructure. The *N. langsdorffii*+*N. forgetiana* cluster is sister to *N. alata*, another species with a haploid chromosomal complement of *n* = 9. The N-J tree also shows that *N. longiflora* (*n* = 10) and *N. bonariensis* are not closely related to one another, nor are they closely related to the major cluster containing *N. langsdorffii*, *N. forgetiana*, and *N. alata*. Dominant HR-mediated resistance to *P. tabacina* appears to be expressed by species in both clusters of *Nicotiana* section *Alatae*.

The AFLP data matrix was also examined by PCoA, a multivariate ordination method used to visualize relationships within a data set. In PCoA, pairwise distances computed between individual variables are projected as coordinates upon a set of derived orthogonal axes, and similar cases group together. The distribution of the 18 *Nicotiana* accessions from Section *Alatae* is shown in Figure 2B. Eigenvalues for the first three PCO axes were 19.681, 14.502, and 10.058, respectively, representing 44.241% of the total variation. The major clusters from the N-J cluster analysis are retained, and PCoA shows the relationships between the different accessions more clearly than does the N-J tree. The close similarity between S-4-1 and S-4-5 is readily apparent, as are the outlying positions of S-4-8 in cluster A and S-4-3 in cluster B-2. The only real difference between the two analyses of the AFLP data is that subclusters A-1 and A-2 appear not to be resolved in the PCoA plot of axes 1 and 2, although these two groups are well separated on axis 3 in a three-dimensional plot (not shown).

## Discussion

### Linkage mapping in *N. langsdorffii*

Detection of linkage to molecular markers, and subsequent linkage mapping, are the prerequisites for map-based cloning of a candidate plant disease resistance gene. Because *N. langsdorffii* is genetically uncharacterized, we chose to use AFLPs to construct a basic linkage map framework onto which we would then map *NlRPT* and other classes of molecular markers such as SSRs, CAPS, and ISSRs. AFLP is a PCR-based technique that combines RFLP with specific amplification of small genomic DNA fragments (Vos et al., 1995). AFLPs have wide utility in plant genetics, particularly in the areas of linkage map construction, genetic diversity analyses, population studies, and cultivar identification (DNA fingerprinting). Microsatellites, or SSRs, are highly variable loci that are distributed widely throughout eukaryotic genomes (Tautz, 1989) and have proven to be informative genetic markers in plants (Phillips and Vasil, 2001; Varshney et al., 2005). SSRs are co-dominant and generally display a high level of polymorphism in outcrossing species (Morgante and Olivieri, 1993; Koreth et al., 1996). We also employed other types of polymorphic amplifiable molecular markers, such as SCAR (Albani et al., 2004), COSII (Wu et al., 2006), ISSR (Sankar and Moore, 2001), and TRAP (Hu and Vick, 2003) markers in construction of the first linkage map of the *N. langsdorffii* genome.

While our choice of mapping population structure may seem novel, it allowed us to collect allelic segregation data for several different types of molecular markers and integrate the data in a common format. We refer here to the set of progeny used in the mapping of *NlRPT* as a “modified backcross” population, but these plants are actually F_1_ hybrids between *N. langsdorffii* and *N. forgetiana*. What gives this population genetic utility is the fact that the female parent was a hybrid between two distinct genotypes of *N. langsdorffii*, S-4-1 and S-4-4. Like a standard backcross population, genetic linkage estimates were calculated from meiotic recombination that occurred in the S-4-1 × S-4-4 F_1_ parent only. *N. forgetiana* is closely related to *N. langsdorffii*; the two species share the same number of chromosomes (2*n* = 18), and are interfertile. In a genomic sense, however, *N. forgetiana* is distinct enough from *N. langsdorffii* such that we could readily identify fluorescent AFLP peaks unique to either of the two parental *N. langsdorffii* accessions (S-4-1 and S-4-4) that were not present in *N. forgetiana* S-11-2. This approach allowed us to score AFLPs that originated from either S-4-1 or S-4-4 without having to be concerned with interfering peaks from the *N. forgetiana* parent. We used the same strategy to score allelic DNA fragments amplified with primer pairs for SSR, COSII, SCAR, and CAPS markers which, unlike AFLPs and TRAP markers, generally show co-dominant inheritance. The expected segregation ratios for all markers and the resistance gene *NlRPT* were 1:1 in this population.

We initially used BSA to identify AFLPs that were linked to *NlRPT*. BSA is a powerful method for identifying molecular markers that show association with a gene of interest or a specific region of the genome (Michelmore et al., 1991). By pooling DNA from individuals with the same phenotype, only the region of the genome surrounding a specific genetic locus is compared for the two parental alleles. BSA has been widely used in many crop species for detecting markers linked to single genes conferring disease resistance (Asnaghi et al., 2003; Hyten et al., 2009), and has recently been shown to have utility for mapping QTL for clubroot resistance in *Brassica napus* (Werner et al., 2008). Due to the dominant inheritance of *NlRPT* and the composition of the two susceptible bulks (S#1 and S#2), only markers linked in *cis* would be detected by BSA. All four of the AFLPs that showed association with *NlRPT* by BSA also showed genetic linkage to *NlRPT* on the map (Figure 1). We identified a fifth peak (E36M49_261) that showed association with the resistance gene, but because the primer combination E36M49 was used only in the BSA screening, it does not appear on the linkage map. Another 12 AFLPs were found that met most but not all of the screening criteria given above (i.e., absent in one S bulk but present in the other, drastically reduced but not absent in the two S bulks, etc.). Of these, only E41M50_198 was linked to *NlRPT*.

We identified 10 COSII markers, originally mapped in the tomato genome (Mueller et al., 2005; Wu et al., 2006), that were polymorphic between *N. langsdorffii* S-4-1 and S-4-4. Markers of this general class were included in an attempt to detect any macrosynteny that might exist between *S. lycopersicum* and *N. langsdorffii*. One of these COSII markers (At2g34560) did not show linkage to any of the other marker loci on the *N. langsdorffii* map. The other 9 primer pairs gave segregation data for 10 genetic loci – At5g37360 had two bands that segregated independently following digestion of the PCR products with *Dpn*II. The ten COSII markers mapped to single loci on the *N. langsdorffii* linkage map, with three on LG4, three on LG8, two on LG5, and one each on LGs 2 and 3. The three COSII markers on LG4 (At1g30580, At1g60640, and At4g21520) all map to chromosome 2 in tomato. The marker locus order is similar between the two maps, although the recombination distances are quite different. A fourth marker, At2g34560, also from tomato chromosome 2 was polymorphic in our mapping population but was not included (see above). Of the three COSII markers mapped to LG8, two of them (At2g16920 and At5g64350) map to chromosome 1 in tomato. The third COSII marker on LG8 is At5g37360, which maps to a locus on tomato chromosome 4. This discrepancy is probably explained by the fact that we mapped a secondary locus (designated “B”) for this marker that was not detected in tomato, and therefore does not appear on the tomato linkage map. LG5 has two COSII marker loci, both of which map to chromosome 3 in tomato. Based on these highly conserved COSII markers, LGs 4, 5, and 8 in *N. langsdorffii* may correspond, at least in part, to tomato chromosomes 2, 3, and 1, respectively. This assessment is based on very limited data, and further mapping of characterized COSII markers on the *N. langsdorffii* genome will be needed to define the relationships between the 12 chromosomes of tomato and the 9 chromosomes of *N. langsdorffii*. Three of the COSII markers mapped in *N. langsdorffii* were also mapped in two other species of *Nicotiana*; At1g30580, and At2g16920 were mapped in *N. tomentosiformis*, and At5g37360 was mapped in this species and also in *N. acuminata* (Wu et al., 2010). Because these three markers mapped to loci on three different chromosomes in *N. tomentosiformis*, their utility for interspecific comparative genomics is limited at present.

Segregation distortion (SD), defined as the statistically significant deviation of genotypic frequencies from Mendelian expectation (Lu et al., 2002), was observed for a subset of the marker loci. Twenty-nine of the most highly distorted (*p* < 0.01) loci were removed from the data set and the map was recalculated. All of these were AFLP markers, and it is possible that the observed distortion could be due to difficulties encountered in scoring the peaks, or to differential peak amplification from some progeny DNA during PCR. In the final version of the linkage map, 61 (22.9%) of the marker loci exhibited SD (*p* < 0.05) and were distributed over all LGs except LGs 4, B, and C. We detected three segregation distortion regions (SDRs) on LGs 1, 5, and 9 that together comprised 38 loci covering 45 cM (indicated in bold in Figure 1). The SDR on LG1 (11 loci and 23 cM) is skewed toward the S-4-4 (male) parent, while the SDRs on LG5 (18 loci, 16.2 cM) and LG11 (9 loci, 5.8 cM) both favor alleles from S-4-1 (the female parent). Overall, 62% of the distorted loci are located within SDRs. SD is a common feature of plant genetic linkage maps; it is frequent in progeny derived from interspecific crosses, and distortion tends to increase with increasing numbers of meioses in intraspecific crosses (Lu et al., 2002; Thoquet et al., 2002). The presence of SDRs can indicate that some genetic or physiological mechanism, such as gametophytic or zygotic selection, may be the underlying cause of the observed SD. In *N. langsdorffii*, we can only speculate as to the existence of the three SDRs. Both of the original parents of the mapping population, S-4-1 and S-4-4, are highly inbred, and we have observed that S-4-1 has much lower seed set, and both poorer and delayed germination when compared to S-4-4 (unpublished). Deleterious alleles originating from S-4-1 for one or more of these traits could account for the largest SDR, which is on LG1 and is strongly skewed toward alleles from S-4-4.

The linkage map and molecular markers developed for the *N. langsdorffii* genome could be useful tools for map-based cloning of *NlRPT*. The *NlRPT* locus on LG3 is flanked most closely by three cis-linked AFLP markers that originated in S-4-4, the source of this resistance gene. To make these markers useful for isolating *NlRPT*, AFLP fragments E33M48_292, E41M50_198, and E39M51_224 would need to be cloned and sequenced to convert them into SCAR markers. At present, E41M50_198 is the closest locus to *NlRPT*, and it is ∼2.3 cM distant. A much larger F_2_ mapping population and focused screening of the COSII marker collection would be needed to identify flanking marker loci that are tightly linked (<1 cM) to *NlRPT*. Additional genomic resources, such as an extensive EST collection and draft genome sequence, would be very useful for isolating important genes from *N. langsdorffii*. Like many species of *Nicotiana*, *N. langsdorffii* (a diploid) has a large genome, in this case ∼4300 Mbp (Narayan, 1987), which is ∼15% smaller than that reported for the allopolyploid *N. tabacum* (Leitch et al., 2008).

### Genetic relationships within *Nicotiana* section *Alatae*

We also used AFLP marker data in a phenetic clustering study to examine the relationships between 14 *N. langsdorffii* accessions and four other closely related species in *Nicotiana* section *Alatae*, 11 of which expressed dominant resistance to *P. tabacina*. Previous studies in plants have demonstrated that AFLPs are particularly informative in closely related taxa, where potential errors in genetic distance estimates due to fragment homoplasy are minimized (Crawford and Mort, 2004; Parks et al., 2006; Krichen et al., 2008). In their study of relationships within *Echinacea*, Kim et al. (2004) used an automated DNA sequencing instrument for fluorescent AFLP detection, demonstrating that individual fragments are amplified very reliably in this adaptation of an already robust technology. We used the same instrument (Beckman Coulter CEQ8000) in this study, and analysis of our data showed conclusively that AFLPs are highly informative in *N. langsdorffii* and could be used for genetic mapping and to infer genetic relationships within section *Alatae*. The N-J tree computed from the AFLP data clearly resolved the 14 accessions of *N. langsdorffii* into two well-defined clusters (A and B), with *N. forgetiana* as the sister species to *N. langsdorffii* (Figure 2A). The conclusions drawn from the N-J analysis are fully supported by PCoA of the same AFLP data set. In PCoA, the relationships between the plant accessions can be displayed spatially in either 2- or 3-dimensional plots. In our analysis, the relative positions of *N. langsdorffii* accessions S-4-3 and S-4-8 can be visualized more clearly than in the N-J tree, as can the relationships between the 14 *N. langsdorffii* accessions and the other four species of *Nicotiana* in section *Alatae* (Figure 2B).

Previous experience with AFLP data from wild and cultivated *Sinningia speciosa* (the florist’s gloxinia of horticulture) showed that clustering methods (N-J, UPGMA, and PCoA) applied to binary AFLP data grouped plant accessions by geographical origins, where known, and made it possible to assign unknown accessions to regions of southeastern Brazil based on cluster assignments (D. Zaitlin, unpublished). In the purple coneflower (*Echinacea angustifolia*), a N-J tree derived from AFLP data for 10 US populations along a north-south climatic gradient also displayed a very strong geographical association for all clades (Still et al., 2005). The only *N. langsdorffii* accessions for which we have collection data are those designated MO-9 (R and S), MO-10, and MO-11, which are from the adjacent towns of Papanduva (MO-9) and Major Vieira (MO-10 and MO-11) in the southern Brazilian state of Santa Catarina. These four accessions grouped together in cluster B along with S-4-1, S-4-3, and S-4-5, the three accessions that form compatible interactions with *P. tabacina*. According to Goodspeed (1954), *N. langsdorffii* occupies a large area in South America, extending from the Brazilian states of Rio de Janeiro and southern Minas Gerais in the east, southwest through São Paulo, Paraná and Santa Catarina into eastern Paraguay and northeastern Argentina. Thus, for *N. langsdorffii*, the two major clusters could very well represent distinct lineages that originated from different regions within the geographical range of this species.

Our present knowledge of dominant HR-mediated resistance to *P. tabacina* indicates that this specific reaction is limited to species in *Nicotiana* section *Alatae*. We previously identified this form of host-pathogen incompatibility in ∼70% of the *N. langsdorffii* accessions that we tested (Zhang and Zaitlin, 2008). Controlled inoculations of the available accessions of *N. forgetiana* (*n* = 2), *N. alata* (4), *N. longiflora* (5), and *N. plumbaginifolia* (2) revealed that these species, like *N. tabacum*, are fully permissive hosts for the blue mold pathogen. Plants of a single accession of *N. bonariensis* expressed a necrotic lesion reaction when inoculated with asexual sporangia of *P. tabacina*. This reaction was indistinguishable from those observed on resistant accessions of *N. langsdorffii* in terms of appearance, time of onset, and the fact that it completely prevented the pathogen from colonizing the inoculated leaves (not shown). If this resistance is due to expression of an orthologous gene present in *N. bonariensis*, the N-J tree indicates that resistance could have evolved in the common ancestor of these two species. Of course, further genetic studies and cloning of the gene will be necessary to determine if it is orthologous to *NlRPT* from *N. langsdorffii*.

### Cross-species utility of *N. langsdorffii* SSR markers

We were able to design acceptable primer pairs flanking SSR motifs for 98 of the genomic clones (out of >300 sequenced). Of these, 30 gave length polymorphisms that distinguished the parents of our *N. langsdorffii* mapping population (S-4-1 and S-4-4) from one another, and 25 of them map to loci on the molecular linkage map (Figure 1). Our initial screening panel of genomic DNA samples also included two accessions each of *N. alata* and *N. longiflora*, *n* = 9 and *n* = 10 species in section Alatae, respectively. Approximately 60% of the SSR primer pairs directed amplification of discreet genomic fragments from these two species. Thirteen detected polymorphism in *N. alata*, and 20 in *N. longiflora*, indicating their potential utility as genetic markers in species related to *N. langsdorffii*. We also screened 20 of these primer pairs, eight of which (GIS_A4, GIS_B3, GIS_D1, A113, A118, A159, B162, and C156) were used for mapping in *N. langsdorffii*, against six accessions of the Australian species *N. benthamiana* (section Suaveolentes). Unexpectedly, all gave discreet amplification products from *N. benthamiana* genomic DNA. This result, while not definitive, supports the genomic hybridization experiments of Chase et al. (2003), which show that an ancestral species from section Alatae was one of the progenitors of sect. Suaveolentes (all are of polyploid origin), a concept originally proposed by Goodspeed (1954).

## Conclusion

The research presented herein shows conclusively that molecular markers are useful for constructing a linkage map of the *N. langsdorffii* genome and for detecting associations between marker loci and known R-genes. BSA, using pools of F_2_ plants that were susceptible to *P. tabacina* infection, was an effective strategy for identifying AFLP markers linked to the resistance gene *NlRPT*. We also showed that the structure of the mapping population (a “modified backcross”) allowed all markers, even those with null alleles, to be scored in the same way, making calculation of the linkage map very straightforward. Several AFLPs and one SSR marker locus showed linkage to *NlRPT* at the distal end of LG3. For map-based cloning of this gene, at least two of the flanking AFLP markers (E33M48_292 and E41M50_198) would need to be converted into more user-friendly (i.e., SCAR) markers. As is common in genetic linkage mapping, some of the marker loci showed evidence of SD. The SD observed in this *N. langsdorffii* mapping population was largely confined to three SDRs on LGs 1, 5, and 9, accounting for 38 marker loci and 45 cM (4.2% of the total map length). In the largest SDR (23 cM on LG1), marker alleles are strongly skewed toward the S-4-4 parent, indicative of a possible physiological effect.

Our study also demonstrated that AFLP-based phenetic analyses can be used to elucidate relationships within *N. langsdorffii*, and between this taxon and related species in *Nicotiana* section *Alatae*. PCoA (Figure 2B) strongly indicated that there are two or three genetic lineages represented in the 14 accessions of *N. langsdorffii* included in this study, which could be related to their geographical origins. HR-mediated resistance to *P. tabacina* is present in both major clusters (A and B-2), and also in the related species *N. bonariensis*.

A recent AFLP-based analysis of *N. benthamiana* (Goodin et al., 2008) indicated that most of the accessions used in research have very limited genetic diversity. However, one accession (USDA PI#555684) included in the distance-based diversity study is a much larger plant that is morphologically close to the type collection that proved to be distinct from all of the research accessions (see Figure 3 of Goodin et al., 2008). Our limited screening of the *N. langsdorffii* SSR marker primers showed that 6 out of 20 detected polymorphism within the group of *N. benthamiana* accessions, and in all cases PI#555684 was different from the five research accessions, which were identical (data not shown). Thus, many of the SSR markers developed for the *N. langsdorffii* genome are of potential use for genetic studies in *N. benthamiana* and its close relatives in section *Suaveolentes*.

## Authors Contributions

Shouan Zhang participated in the design of the mapping strategy, generated much of the AFLP and molecular marker data, and drafted and proofread the manuscript. Muqiang Gao assisted with data generation and collection, and helped with the linkage mapping. David Zaitlin directed the project, developed the molecular markers and mapping population, generated allelic marker data, and wrote the manuscript.

## Conflict of Interest Statement

The authors declare that the research was conducted in the absence of any commercial or financial relationships that could be construed as a potential conflict of interest.

## Footnotes

^1^http://www.keygene.com/home/index.php

^2^http://frodo.wi.mit.edu

^3^http://eu.idtdna.com/site

^4^http://solgenomics.net
